# A Critical Review of Dr. W. B. Ames’ Article on Atmospheric Pressure, Etc., as Published in the July Number of the Independent Practitioner

**Published:** 1885-09

**Authors:** C. H. Land

**Affiliations:** Detroit, Mich.


### A CRITICAL REVIEW OF DR. W. B. AMES' ARTICLE ON ATMOSPHERIC PRESSURE, ETC., AS PUBLISHED IN THE JULY NUMBER OF THE "INDEPENDENT PRACTITIONER."

BY DR. C. H. LAND, DETROIT, MICH.

The entire force of the argument of Dr. Ames is concentrated on what he asserts is a powerful pressure of the atmosphere. This it seems to me is an absurdity. For how can there be any manifestation of atmospheric pressure without an air space and some exhausting means. These ideas seem to pervade almost the entire dental profession, and, as a consequence, if one goes into any dental laboratory, not excepting those of the colleges, he will find the ubiquitous air chamber staring at him as an emblem of stupidity. It may be seen on signs, on cards, bill heads, letter heads, and in the newspapers. The beautiful little heart, shield, or star, is the vehicle of last resort for the suction-plate dentist, who, for the want of a better training in the proper method of inserting a relief, places a leaden heart in the most rigid portion of the palatine surface, for no other reason than because he saw his preceptor do it. In order to enable Dr. Ames and others to test the extent of this pressure, let me suggest the following:

1st. Take a glass chimney, such as is used for the ordinary student's lamp, get a two-pound weight, cover it with rubber dam, press the large end of the chimney on the weight, and with the mouth exhaust the air, and it will be demonstrated that the power exerted by the tongue as an exhausting means, under the most favorable conditions, will not represent a retaining force greater than about one pound to the square inch.

2d. Take a piece of leather one and one-half inches in diameter, fasten a string in the centre, soak it in water, then press it close to a six-pound weight, and a retaining force of two pounds' adhesion to the square inch will be manifested, a contact force one hundred per cent. greater than where the tongue is employed as an exhausting means. In this latter experiment we have a manifestation of capillary attraction, induced by the adhesive attraction that all fluid substances have for solid bodies, to such an extent that the water will act against its own gravity, as illustrated in a sponge. It will force the air out of the intermediate spaces and take its place. In this way an equilibrium is established, by which the atmosphere meets with a counter-resistance sufficient to completely balance the weight, consequently there is no pressure. The saliva, or fluids of the mouth, cause the denture to adhere with a retaining force of about two pounds to the square inch, forcing the air out from beneath the denture, and the tissues of the mouth fill up all the empty spaces. From these facts it should become evident to the most casual observer, that the term atmospheric denture is a misnomer. However, if we could utilize the pressure of the atmosphere through the agency of an exhausting pump, even with one-pound pressure to the square inch, there could be no better means devised for maintaining a denture. There would be this practical advantage: the air pressing equally on all sides would cause the denture to be held firmly in position from all directions, no matter what the shape of the mouth, while a capillary contact will slip or slide on the angles, and only represent its maximum retaining force on horizontal surfaces. For this reason angular mouths are the most difficult to fit, especially if they are rigid and dry.

The scientific facts that I wish to present to the profession may be enumerated as follows:

1st. That artificial dentures are not maintained by the pressure of the atmosphere, and, if they were, that the tissues of the mouth would not tolerate even one pound pressure to the square inch.

2d. That they are maintained through the agency of an intermediate fluid, which represents a retaining force of about two pounds to the square inch on horizontal surfaces.

3d. That air spaces should be provided, not for the purpose of forming a partial vacuum, but simply as a relief to the dense portions of the maxillary bone and the rigid parts of the palatine arch. This relief, in a mouth where the alveolar ridge is comparatively soft, should cover four-fifths of the interior surface of the mouth, and be just sufficient in depth to make the outer fifth become the most positive in contact. The mouth being of a yielding nature, it soon conforms to this central relief, and, as a result, we secure a balanced denture, having established the first positive contact at the greatest diameter of the mouth. A mouth that is generally rigid, representing a surface so closely allied to a rigid denture, would indicate a balanced contact. Consequently, a good impression is about all that is necessary to secure perfect results. On the contrary, a mouth that presents certain rigid parts in combination with soft or flexible tissues is an indication of the necessity of relieving the denser portions on all horizontal surfaces, in this way producing a balanced contact. When properly manipulated this should reward the dentist with the utmost possibilities of success in the application of a rigid denture to the dental arch.

